# The effect of physical exercise and cognition-orientated interventions on post-stroke cognitive function: Protocol for an overview of reviews

**DOI:** 10.1371/journal.pone.0318567

**Published:** 2025-01-29

**Authors:** James Smith, Philip Nagy, David Tod, Carol Holland, Hannah Jarvis

**Affiliations:** Faculty of Health and Medicine, Lancaster University, Lancaster, United Kingdom; University Hospital Cologne: Uniklinik Koln, GERMANY

## Abstract

**Background:**

Strokes are becoming more common, and with improving survival rates, the prevalence of stroke survivors has increased. Almost half of chronic stroke survivors are cognitively impaired, and healthcare services are struggling to manage these patients, leaving some feeling “abandoned”. Several systematic reviews have investigated the effect of physical exercise and cognition-orientated interventions on post-stroke cognitive impairment, and have produced conflicting findings, making it difficult for clinicians and guideline producers to make evidence-based decisions. This overview of reviews aims to provide a comprehensive overview of systematic reviews investigating the effect of physical exercise and cognition-orientated interventions on post-stroke cognitive function, assess methodological quality and certainty of evidence, and identify sources of discordance between these reviews.

**Methods:**

Eight databases–Embase, Medline, CINAHL, Psycinfo, SPORTDiscus, The Cochrane Database of Systematic reviews, Epistemonikos, and Scopus–plus grey literature sources will be searched. The eligibility criteria include systematic reviews of trials that included an adult stroke population and investigated physical exercise and/or cognition-orientated interventions. Only reviews that assessed at least one of the DSM-5 neurocognitive domains will be included. Screening, data extraction, and quality appraisal will be conducted by two independent reviewers. Methodological quality, certainty of evidence, and primary study overlap will be assessed using the AMSTAR-2, GRADE, and GROOVE tools, respectively. Interventions will be grouped into exercise, cognition-orientated, and combined interventions, and findings will be synthesised narratively. Heterogeneity assessment will be conducted to identify factors causing discordance between reviews.

**Discussion:**

The findings of this overview will allow decision makers to make evidence-based decisions, stratified by methodological quality and certainty of evidence. Heterogeneity assessment may identify factors causing discordance between systematic reviews, which could inform the design of future studies.

**Trial registration:**

**Registration:** PROSPERO CRD42024534179.

## Introduction

Stroke is the third-leading cause of death and disability in the world [1]. The incidence of stroke has been steadily increasing over the past two decades, and is projected to continue until at least 2030 [2]. However, more people are surviving strokes than ever before [3], resulting in an ever-growing population of stroke survivors, of which 44% will experience cognitive impairment between 2 and 6 months after stroke [4]. The UK Stroke Association has stated that “neuropsychological support for stroke survivors is in a relative state of crisis”, with stroke survivors receiving an average of 5.3 minutes of psychological treatment per day from community services and waiting times being an average of ten weeks [5, 6]. Clearly, the current organisation of stroke rehabilitation services are unable to provide stroke patients with recommended amounts of treatment, and patients are now feeling “abandoned” [5].

Cognition-orientated interventions, that is any intervention that directly or indirectly targets cognitive functioning as opposed to interventions that focus primarily on behavioural, emotional, or physical function [7], and physical exercise have both been shown to improve post stroke cognitive function in humans [8–12]. The mechanisms through which physical exercise improves cognitive function after stroke are complex, with many different body systems being affected. A review by ploughman et al. found that, in rodents, levels of neurotrophic factors (brain-derived neurotrophic factor and insulin-like growth factor-1) in the brain increased in response to physical exercise [13]. Also in rodents, reduced lesion volume, protection against oxidative stress, reduced inflammatory markers, reduced cell death [14]; increased cerebral blood flow [15]; blood-brain barrier repair [16]; and modulation of mitochondrial health through myokines [17], have also been observed following physical exercise. This list is not exhaustive but demonstrates the complexity and multifarious nature of the underlying mechanisms. Exercise may also be used to ‘prime’ behavioural interventions [18]. When used immediately before a behavioural intervention, individuals will experience the positive benefits of exercise, allowing subsequent interventions to have greater benefit [19]. Exercise priming is a relatively new concept, but clinical trials have found that exercise combined with other interventions may be more beneficial than exercise alone for post-stroke cognitive function [19–21].

Since being named as the top research priority for stroke in 2012 [22], the volume of research investigating post-stroke cognitive impairment (PSCI) has grown significantly. Since 2020, there have been several systematic reviews (SRs) published investigating both physical exercise [8, 10, 23–28], and cognition-orientated interventions [11, 12, 29–31]. However, conflicting findings and variation in methodological quality between these reviews makes it difficult for clinicians, guideline producers, and commissioners to make evidence-based decisions. Furthermore, given the number of reviews published in a short period of time, it is unclear if these reviews are contributing new information to the evidence-base, or simply reanalysing the same primary studies.

The aims of this overview of reviews are: (1) to provide a comprehensive overview of SRs that have investigated the effect of physical exercise and/or cognition-orientated interventions on any domain of cognitive function in adult stroke survivors, due to the large number of SRs already published; (2) to assess the methodological quality and certainty of evidence to allow decision-makers (i.e., clinicians and commissioners) to stratify decisions based on the best available evidence; (3) to assess primary study overlap between SRs; (4) to identify factors causing discordance between systematic reviews, as to date no studies have attempted to identify moderating factors causing discordance between SRs within this field, which could further the understanding of the mechanisms behind these interventions, and subsequently inform the design of future studies; (5) to collect and summarise data on adverse events, to assess the safety of exercise and cognition-orientated interventions in a stroke population.

## Methods

### Protocol development

An overview of reviews–also commonly referred to as an overview, overview of systematic reviews, review of reviews, review of systematic reviews, or umbrella review–aims to collate, assess, and synthesise evidence from multiple systematic reviews on a specific topic [32]. The current protocol was developed and reported in accordance with the Preferred Reporting Items for Systematic Reviews and Meta-Analyses Protocols (PRISMA-P) 2015 (S1 Appendix) [33], as well as the preferred reporting items for overviews of reviews (PRIOR) statement [32]. This protocol has been registered with the International Prospective Register of Systematic Reviews (PROSPERO) database (Registration number: CRD42024534179).

### Eligibility criteria

#### Types of studies

Only systematic reviews (SRs) of randomised controlled trials (RCT) or non-randomised controlled trials, that meet the definition outlined in the PRIOR statement [32], with or without meta-analysis, will be included in this overview. Network meta-analyses will be excluded, unless pair-wise meta-analysis was also conducted. No restrictions will be placed on date of publication. No language filters will be applied to the search strategy; and non-English language publications will be machine translated using Google Translate to allow eligibility assessment and data extraction, to minimise language bias [34]. SRs of qualitative studies (e.g., meta-ethnography) and non-systematic reviews (e.g., narrative reviews) will be excluded. If a systematic review (SR) was an update of a previously published review (addressed the same research question and contained 100% of the primary studies in the original review), only the updated review will be included.

#### Types of participants

Reviews will be included if they synthesised data from adult participants (aged >18) with a history of stroke, according to the World Health Organization definition [35], and reported these data separate from other populations. Participants with and without cognitive impairment at baseline will be included. SRs that included only a stroke population with aphasia and/or hemispatial neglect will be excluded. Although cognitive impairment commonly occurs alongside aphasia and hemispatial neglect, these symptoms alone do not constitute a cognitive impairment [36, 37], and can make cognitive assessment, using the most widely used tools, impossible [38].

#### Types of intervention

Reviews that investigated an exercise intervention, cognition-orientated intervention, or a combination of both will be included. Exercise interventions are defined as planned, structured, and repetitive bodily movement done to improve or maintain a component of physical fitness [39], (e.g., walking programmes, circuit training programs). Cognition-orientated interventions are defined as interventions that directly or indirectly target cognitive functioning as opposed to interventions that focus primarily on behavioural, emotional, or physical function (e.g., computerised cognitive training) [7]. Combined interventions are defined as interventions that contained components that met the definition of both exercise and cognition-orientated interventions (e.g., psychomotor dual-task training). Interventions that combined exercise and/or cognition-orientated interventions with other types of interventions, not typically implemented as part of standard care (e.g., pharmacological, acupuncture, electrical brain stimulation), will be excluded. SRs investigating virtual reality interventions will be eligible, provided all interventions included met either exercise or cognition-orientated intervention definitions. Virtual reality interventions that were primarily implemented to improve motor outcomes will be excluded, even if cognitive outcomes were measured.

#### Types of comparisons

Reviews that compared exercise or cognition-orientated interventions against either standard care, sham/ placebo interventions, or passive control will be included. A broad comparator criterion was used to ensure a comprehensive overview of SRs. Reviews that have compared physical exercise and/or cognition-orientated interventions against an inappropriate control, that is, other active forms of treatment not routinely implemented in standard care (e.g., pharmacological, acupuncture, electrical brain stimulation), and not separated these from eligible control conditions will be excluded.

#### Types of outcomes

To be included in this overview, reviews must have synthesised data on cognitive outcomes, as a primary or secondary outcome, using a validated measure of global cognitive function, or any of the six neurocognitive domains (complex attention, executive function, learning and memory, language, perceptual-motor, social cognition) named in the DSM-5 [40].

#### Intervention sub-groups

The primary intervention groups (physical exercise, CO, and combined) will be further divided into sub-groups. Physical exercise interventions will be divided into aerobic, resistance, other, and mixed. Exercise recommendations for stroke survivors include both aerobic and resistance training [41], justifying their use as sub-categories. However, other types of exercise, such as yoga and Pilates, are commonly implemented for stroke survivors, and require their own sub-group. It is common for an exercise program to incorporate different types of exercise; for example, aerobic and resistance training, so a mixed exercise sub-group was used to identify these training programs.

The American College of Sports Medicine definition of aerobic exercise–any exercise that uses large muscle groups, can be maintained continuously and is rhythmic in nature–will be used in this overview [42]. Note that the final part of this definition (“and uses aerobic metabolic pathways to sustain the activity”) is not included to avoid excluding studies that did not measure metabolism, but otherwise met the definition.

Resistance exercise is defined as any form of periodic exercise whereby external resistance provide progressive overload to skeletal muscles in order to make them stronger (e.g., resistance band exercise program) [43]. Other exercise is defined as any exercise intervention that met the definition of physical exercise by Caspersen et al. (1985) [39], but did not meet the definitions of aerobic or resistance exercise (e.g., tai-chi). Mixed exercise is defined as any exercise intervention that was composed of elements that met the definitions of at least two physical exercise sub-groups.

Cognition-orientated interventions will be divided into: restorative, compensatory, and mixed approaches. Restorative approaches aim to regain lost cognitive function through repetition and practice of tasks (e.g. computer-based cognitive training) [44]. Compensatory approaches use strategies and tools that alleviate the impact of cognitive impairment, without the expectation of improvement in cognitive functioning (e.g., memory aids, mnemonics) [44]. Interventions that combined restorative and compensatory approaches will be grouped as mixed cognition-orientated interventions.

Combined interventions will be divided into concurrent and consecutive. A concurrent-combined intervention applies an exercise and cognition-orientated intervention simultaneously (e.g., psychomotor dual-task performance). A consecutive-combined intervention applies a discrete block of either exercise or cognition-orientated intervention, followed by another discrete block of the other (e.g., aerobic exercise followed by computerised cognitive training).

### Search strategy

#### Search terms

A comprehensive search strategy, containing both controlled vocabulary and free-text search strings, was developed by adapting search strategies of pre-existing SRs [26, 45, 46]. Additional search terms were identified using comprehensive pearl growing [47], using the results of a preliminary search of google scholar as the starting point. SR search filters [48–50], specific to each database, were used to increase the specificity of the search strategy. Controlled vocabulary and syntax were adapted to each database. The search strategy was reviewed by an information specialist during development. The complete search string for Embase (Ovid) can be found in S2 Appendix.

#### Information sources

The following eight databases will be searched from inception to present by one reviewer (JS): Embase (Ovid), Medline (Ovid), CINAHL (EBSCO), APA Psycinfo (EBSCO), SPORTDiscus (EBSCO), The Cochrane Database of Systematic Reviews, Epistemonikos, and Scopus. No limits will be applied to searches, other than stroke group only on the Cochrane Database of Systematic Reviews and SRs only on Epistemonikos. Citation tracking will also be used to identify records not collected through electronic database searches. Backwards citation tracking will be conducted by manually searching the bibliographies of all included studies, and forwards citation tracking will be conducted using Scopus [51]. The PROSPERO register will be searched to identify relevant ongoing reviews and completed reviews that may not have been captured through database searches.

Grey literature will be searched using ProQuest (Dissertations & Thesis and Publicly Available Content Databases). Authors of conference abstracts will be contacted for full-text availability, if not already identified through database searches. Any author named on two or more SRs included in this overview will be contacted to identify any potential records that have not been retrieved by the search strategy.

#### Selection process

The results of electronic database searches will be uploaded to EndNote 20 (https://endnote.com) for de-duplication, using the method outlined by Bramer et al. [52], then exported to Rayyan for screening [53]. The titles and abstracts of all identified SRs will be screened by two independent reviewers. Conflicts will be resolved through discussion of the two conflicting reviewers, until consensus is achieved, or a third reviewer if consensus cannot be reached. The inter-reviewer agreement, calculated using Cohen’s Kappa, will be reported.

Following title and abstract screening, the full-text manuscripts and supplementary materials of the remaining studies will be sought. The full-texts will be screened by two independent reviewers. Conflicts will be resolved through discussion of the two conflicting reviewers, until consensus is achieved, or a third reviewer if consensus cannot be reached. Inter-reviewer agreement (Cohen’s Kappa) will be reported. A complete list of SRs excluded at this stage will be reported, with their reason for exclusion. In line with the latest PRISMA guidance, a flow diagram will be used to represent the flow of items through the selection process [54].

#### Data extraction and methodological quality assessment

Data extraction and methodological quality assessment will be conducted by two independent reviewers. Discrepancies will be resolved through discussion of the two reviewers, until consensus is achieved, or a third reviewer if consensus cannot be reached.

A bespoke data extraction tool (S3 Appendix) and accompanying codebook were created by JS, then iteratively developed through discussion with the wider research team and pilot testing. Variables extracted from SRs will include: country review was conducted, aims of study, databases searched, date of last literature search, inclusion/ exclusion criteria, intervention type and parameters (i.e., frequency, intensity, time, and type), outcome measures (i.e., mini-mental state examination, Stroop test, etc), included primary studies and their risk of bias, method of synthesis, sample size, population characteristics, primary and secondary results, and adverse events. Any missing data from SRs will first be sought from the respective primary studies, failing this, the review author will be contacted.

The 16-items included in the ‘A Measurement Tool to Assess Systematic Reviews 2’ (AMSTAR 2) [55], were integrated into the data extraction tool to allow simultaneous appraisal of methodological quality. Each of the 16 items are scored as yes, partial yes, or no, with seven items classified as critical domains and given additional weight when classifying the overall quality of the review. Although the classification of critical items can be adapted depending on the context of the study [55], it was deemed appropriate to use the seven original critical domains for this overview. The overall methodological quality of each SR will be rated as: high (0 critical weaknesses, ≤ 1 non-critical weakness), moderate (0 critical weaknesses, ≥ 1 non-critical weakness), low (1 critical weaknesses, with or without non-critical weakness), or critically low (≥ 1 critical weaknesses, with or without non-critical weakness) [55]. The results for each of the 16-domains and the overall confidence rating for all included SRs will be tabulated.

#### Outcomes

All validated outcome measures assessing cognitive function will be included in this overview. Cognitive outcomes will be grouped into global and domain-specific measures. For this overview, global measures will be defined as any assessment tool that assesses at least four of the six neurocognitive domains outlined by the DSM-5 (complex attention, executive function, learning and memory, language, perceptual-motor, social cognition) [40]; and domain-specific measures as any tool that assesses specific neurocognitive domains.

Data on adverse events, defined as any incident that causes harm (impairment of structure or function of the body and/or any deleterious effect arising there from, including disease, injury, suffering, disability and death, and may be physical, social or psychological) to a patient [56], will also be collected.

### Data synthesis

#### Characteristics of included SRs

The characteristics of included SRs will be described narratively and tabulated. This will include information on country of review team, intervention investigated, types and number of primary studies included, control conditions included, population characteristics, method of synthesis, and outcomes investigated.

#### Primary study overlap

Primary study overlap, defined as multiple counting of one or more primary studies included in two or more SRs, which in turn are included in the same overview [57, 58], will be assessed using the Graphical Representation of Overlap for Overviews (GROOVE) tool [59]. The GROOVE tool calculates the corrected covered area (CCA)–the degree of primary study overlap–for a whole matrix of evidence, as well as for each pair of SRs included in the overview [59]. The CCA is reported as a percentage, with 0–5% indicating slight, 6–10% moderate, 11–15% high, and >15% as very high overlap [60]. Additionally, to demonstrate the growth of published SRs and primary studies on this topic over time, a cumulative frequency graph will be developed. As the aim of this overview is to summarise the whole body of SR evidence, no measures will be imposed to mitigate primary study overlap. Due to this, and given the anticipated overlap, data collected from SRs will not be pooled, to avoid giving overlapped primary studies additional statistical weight [60].

#### SRs with narrative synthesis

Reviews will be grouped by their primary intervention (exercise, CO, or combined) and synthesised narratively. There will be no attempts to quantify the findings of narrative syntheses. Other overviews of reviews [61, 62] have used “vote-counting” methods, in which the proportion of primary studies within a narrative synthesis that found a significant treatment effect were calculated. If the proportion of primary studies with significant findings met an arbitrary threshold, such as 80%, the synthesis would be classified as supporting the efficacy of the intervention. However, this can lead to potentially misleading conclusions, as this method fails to consider sample size, risk of bias, and heterogeneity between primary studies. Therefore, the “vote-counting” method will be avoided.

#### SRs with meta-analysis

Where availability of data allows, the standardised mean difference (SMD) reported from each meta-analysis will be converted into Hedges *g* (if not already), using the formula provided by Durlak (2009) [63]. Likewise, mean differences will also be converted to Hedges *g*. Hedges *g* was selected as, compared to Cohen’s *d* and Glass’ △, it is the most accurate effect size estimate regardless of equal variance assumptions being violated or not [64]. Using Hedges *g* as the standardised measure throughout the overview ensures that all effect size estimates are the most accurate, and fair comparisons can be made between meta-analyses. However, this conversion process will not be possible if review authors have not stated how SMD was calculated, not reported standard deviations, or not reported sample sizes. In these instances, the author will be contacted for the required information.

The effect of exercise, CO, and combined interventions on each group of cognitive outcomes will be summarised. Tables will be created to present findings, one for each of the primary interventions–exercise, CO, and combined. The headings of each table will include: intervention sub-group, number of included studies and participants, cognitive domain(s) assessed, AMSTAR 2 rating, GRADE rating, SMD and 95% confidence intervals. As the results will be presented at an outcome level, opposed to review level, multiple outcomes from the same SR may be included in a single table. For example, if a SR synthesised the effects of aerobic and resistance training on global cognition separately, both would be included in the exercise interventions table. SRs that synthesised multiple sub-groups together, for example, all available exercise-based interventions, will be grouped together separately, to ensure these do not get confused with mixed interventions.

Any relevant sub-group or moderation analysis conducted by included SRs will be discussed narratively. From pilot testing, common sub-group analyses were intervention duration, time since stroke, and baseline cognitive function.

#### Certainty of evidence

The certainty of evidence for each meta-analytic outcome included in this overview of reviews will be assessed using the GRADE approach, which is a system for rating the quality of evidence in SRs and other evidence synthesis [65]. For SRs where authors have already conducted GRADE assessments, these will be reassessed (blinded) by the primary author of this overview (JS). If there is a discrepancy between these two GRADE assessments, a third reviewer will resolve the conflict. This method ensures standardised assessments throughout the overview. For papers without GRADE assessment, the primary author (JS) will perform the initial assessment, which will then be reassessed by a second reviewer. Any conflicts will be resolved through discussion, or a third reviewer if consensus cannot be reached. To ensure transparency, the rationale for all upgrades and downgrades of evidence will be reported.

#### Heterogeneity assessment

Potential sources of heterogeneity arising from interventions will be explored through sub-group analysis. Primary intervention groups will be broken down into their sub-groups and results will be synthesised at the sub-group level. Potential sources of heterogeneity arising from baseline population characteristics, comparators, and outcomes will also be explored by comparing the inclusion and exclusion criteria of included SRs, which will be described narratively. A two-tailed Spearman’s rank correlation coefficient will be used to evaluate if methodological quality of SRs, as assessed by the AMSTAR-2, and certainty of evidence, as assessed by GRADE, are associated with effect size estimates. The aim of heterogeneity assessment is to identify potential factors causing discordance between SRs. Meta-regression, will not be used, as primary study overlap will result in additional weighting of overlapped studies [60].

#### Adverse events

The proportion of SRs reporting adverse events will be calculated. All data related to adverse events will be synthesised narratively.

## Discussion

This overview of reviews will provide a comprehensive summary of all SRs investigating the effect of physical exercise and/or cognition-orientated interventions on post-stroke cognitive function. The research investigating PSCI is rapidly expanding; and conflicting findings and variations in methodological quality between SRs in this field are making it difficult to make evidence-based decisions. Given the potential benefits of exercise and cognition-orientated interventions could have on stroke survivors, it is vital that a standardised assessment of methodological quality and certainty of evidence of these SRs is conducted to aid future decision making and to provide recommendations for future research. The findings of this overview will be valuable to clinicians, guideline producers, and commissioners, to help inform evidence-based decision making. Furthermore, by highlighting common areas of poor methodological quality and potential sources of discordance, suggestions can be made to inform the design of future research.

## Supporting information

S1 Appendix Preferred reporting items for systematic review and meta-analysis protocols (PRISMA-P) checklist. A completed checklist of the preferred reporting items for systematic review and meta-analysis protocols.
(PDF)

S2 Appendix Search strategy. The complete search string for Embase (Ovid).
(PDF)

S3 Appendix Data extraction tool. A copy of the data extraction tool that will be used to extract data from systematic reviews.
(XLSX)

## List of abbreviations

AMSTAR 2: A measurement tool to assess systematic reviews 2
CCA: Corrected covered area
GROOVE: Graphical representation of overlap for overviews
PRIOR: Preferred reporting items for overviews of reviews
PSCI: Post-stroke cognitive impairment
RCT: Randomised controlled trial
SMD: Standardised mean difference
SR: Systematic review
SRs: Systematic reviews
UK: United Kingdom
