# Supplementary material for: The effect of physical exercise and cognition-orientated interventions on post-stroke cognitive function: Protocol for an overview of reviews
Source: PLoS One. 2025 Jan 29;20(1):e0318567. doi: 10.1371/journal.pone.0318567 (PMC11778714; doi:10.1371/journal.pone.0318567)
Supplement: S2 Appendix — The complete search string for Embase (Ovid). (PDF) [file pone.0318567.s002.pdf]

Search Strategy for Embase (Ovid)

| | |
|---|---|
| 1. | exp Meta Analysis/ |
| 2. | ((meta adj analy$) or metaanalys$).tw. |
| 3. | (systematic adj (review$1 or overview$1)).tw. |
| 4. | 1 or 2 or 3 |
| 5. | cancerlit.ab. |
| 6. | Cochrane.ab. |
| 7. | embase.ab. |
| 8. | (psychinfo or psycinfo).ab. |
| 9. | (cinahl or cinhal).ab. |
| 10. | science citation index.ab. |
| 11. | bids.ab. |
| 12. | (psychlit or psyclit).ab. |
| 13. | 5 or 6 or 7 or 8 or 9 or 10 or 11 or 12 |
| 14. | reference lists.ab. |
| 15. | bibliograph$.ab. |
| 16. | hand-search$.ab. |
| 17. | manual search$.ab. |
| 18. | relevant journals.ab. |
| 19. | 14 or 15 or 16 or 17 or 18 |
| 20. | data extraction.ab. |
| 21. | selection criteria.ab. |
| 22. | 20 or 21 |
| 23. | review.pt. |
| 24. | 22 and 23 |
| 25. | letter.pt. |
| 26. | editorial.pt. |
| 27. | animal/ |
| 28. | human/ |
| 29. | 27 not (27 and 28) |
| 30. | 25 or 26 or 29 |
| 31. | 4 or 13 or 19 or 24 |
| 32. | 31 not 30 |
| 33. | exp cerebrovascular disorders/ or exp cerebrovascular accident/ or exp cerebrovascular disease/ or brainstem stroke/ or cardioembolic stroke/ or lacunar stroke/ or basal ganglia cerebrovascular disease/ or brain ischemia/ or carotid artery diseases/ or cerebral artery disease/ or cerebrovascular trauma/ or intracranial hemorrhages/ or cerebral hemorrhage/ or pituitary apoplexy/ or subarachnoid hemorrhage/ or brain infarction/ or brain hemorrhage/ or cerebellum hemorrhage/ or massive intracerebral hemorrhage/ or brain arteriovenous malformation/ or brain embolism/ or cerebral thrombosis/ or brain vasospasm/ or vertebral artery dissection/ |
| 34. | exp stroke/ or hemorrhagic stroke/ or ischemic stroke/ |
| 35. | stroke patient/ or stroke survivor/ |
| 36. | (stroke or post?stroke or cerebrovasc$ or cerebral vasc$ or cva$ or apoplex$ or SAH).tw. |
| 37. | ((cerebr$ or cerebell$ or intracran$ or intracerebral) adj5 (isch?emi$ or infarct$ or thrombo$ or emboli$ or occlus$)).tw. |
| 38. | ((cerebr$ or cerebell$ or intracerebral or intracranial or subarachnoid) adj5 (haemorrhage$ or hemorrhage$ or haematoma$ or hematoma$ or bleed$)).tw. |
| 39. | hemiparesis/ or hemiplegia/ or paresis/ or neurologic gait disorder/ or hemiplegic gait/ |
| 40. | (hempar$ or hemipleg$).tw. |
| 41. | 33 or 34 or 35 or 36 or 37 or 38 or 39 or 40 |

Search Strategy for Embase (Ovid)

| | |
|---|---|
| 42. | exp exercise/ or exp physical activity/ or exp kinesiotherapy/ or exp sport/ or exp aerobic exercise/ or exp interval training/ or mobilization/ or endurance training/ or treadmill/ or ergometer/ or treadmill ergometry/ or treadmill exercise/ or locomotion/ or "movement (physiology)"/ or conditioning/ or fitness/ |
| 43. | ((Aerobic or cardio$ or endurance or continuous or interval or high?intensity or low?intensity or treadmill) adj2 (exercis$ or train$ or conditioning)).tw. |
| 44. | (physical activity or aerobics or ergomet$).tw. |
| 45. | 42 or 43 or 44 |
| 46. | exp anaerobic exercise/ or exp resistance training/ or exp circuit training/ or exp functional training/ or muscle strength/ or weight training/ or muscle isometric contraction/ or muscle isotonic contraction/ |
| 47. | ((anaerobic or resistance or circuit or weight) adj2 (exercis$ or train$ or conditioning)).tw. |
| 48. | 46 or 47 |
| 49. | exp cognitive rehabilitation/ or exp cognitive remediation therapy/ or exp cognitive therapy/ or cognitive restructuring/ |
| 50. | (((cognit$ or memory or attention) adj (rehab$ or therap$ or management or stimulation)) or ((cognit$ or memory or attention) adj2 (train$ or re?train$ or intervention or support or aid or group or streteg$ or exercis$ or enhance$ or enrich$))).tw. |
| 51. | 49 or 50 |
| 52. | exp "dual-task performance (test)"/ |
| 53. | ((dual?task$ or cognit$) adj2 (interference or training)).tw. |
| 54. | (divided attention or attention?demanding task).tw. |
| 55. | 52 or 53 or 54 |
| 56. | 45 or 48 or 51 or 55 |
| 57. | exp cognition/ or exp mental function/ or exp cognition assessment/ or exp memory/ or exp brain function/ or exp memory assessment/ or memory disorders/ or language/ or "speech and language"/ or verbal communication/ or language processing/ or language ability/ or exp cognitive defect/ or confusion/ or arousal/ or exp perception/ or orientation/ or attention/ or thinking/ or concept formation/ or recognition/ or decision making/ or awareness/ or exp problem solving/ or comprehension/ or learning/ or montreal cognitive assessment/ or exp cognitive impairment assessment/ or psychologic test/ or exp neuropsychological assessment/ or hemispatial neglect/ or agnosia/ or neurobehavioral manifestations/ or exp cognitive reserve/ or processing speed/ or exp executive function/ or psychomotor performance/ or recall/ or social cognition/ |
| 58. | (cogniti$ or arous$ or orientat$ or attention$ or concentrat$ or memor$ or recall or percept$ or think$ or judg?ment$ or awareness or problem solving or comprehension or brain function or language or communication or decision making or recognition or mental process$ or concept formation or impulsive behavio?r$ or executive function$ or concentrat$ or visuospatial or visual?spatial or reasoning or intelligen$ or planning or agnosia or amnesia or confusion or inattention or neglect).tw. |
| 59. | 57 or 58 |
| 60. | 32 and 41 and 56 and 59 |
